# Potential Impact of Choline Alphoscerate on Depressive Symptoms in Association with Insulin Resistance in Elderly Patients with Type 2 Diabetes

**DOI:** 10.3390/jcm14051664

**Published:** 2025-02-28

**Authors:** Inkuk Lee, Minyoung Lee, Min Heui Yu, Eugene Han, Yong-ho Lee, Byung-Wan Lee, Eun Seok Kang, Bong-Soo Cha

**Affiliations:** 1 Department of Internal Medicine, Yonsei University College of Medicine, Seoul 03722, Republic of Korea; inkuklee@yuhs.ac (I.L.); lmycj@yuhs.ac (M.L.); minheuiyu@yuhs.ac (M.H.Y.); yholee@yuhs.ac (Y.-h.L.); bwanlee@yuhs.ac (B.-W.L.); edgo@yuhs.ac (E.S.K.); 2 Institute of Endocrine Research, Yonsei University College of Medicine, Seoul 03722, Republic of Korea; 3 SENTINEL Team, Division of Endocrinology, Department of Internal Medicine, Yonsei University College of Medicine, Seoul 03722, Republic of Korea; 4 Division of Endocrinology and Metabolism, Department of Internal Medicine, Keimyung University School of Medicine, Daegu 42601, Republic of Korea; eghan@dsmc.or.kr

**Keywords:** depression, diabetes mellitus type 2, glycerylphosphorylcholine, insulin resistance, randomized controlled trial

## Abstract

**Background/Objectives:** The aim of this study was to investigate whether Gliatamin (choline alphoscerate), an acetylcholine precursor originally used to treat dementia, improves depressive symptoms in patients with type 2 diabetes. **Methods:** We conducted a 6-month double-blind, randomized, and placebo-controlled trial involving 49 participants with type 2 diabetes, older than 50 years, and with mild depressive symptoms; 33 and 16 participants received choline alphoscerate (800 mg/day) and a placebo, respectively. **Results:** At 6 months, the Hamilton Depression Rating Scale was significantly decreased in both the choline alphoscerate (15.9 ± 6.5 to 12.6 ± 5.8, *p* = 0.001) and placebo (15.4 ± 4.7 to 10.2 ± 3.9, *p* = 0.004) groups compared with baseline, without inter-group difference (*p* = 0.297). Meanwhile, the choline alphoscerate group showed favorable results in insulin resistance-related parameters at 6 months, including the waist circumference (WC) and the low-density lipoprotein cholesterol (LDL)/high-density lipoprotein cholesterol (HDL) ratio (*p* for inter-group difference vs. placebo = 0.009 and 0.092). Even after adjusting for multiple confounding factors, choline alphoscerate use was associated with an increased odds for reduction in WC (OR 18.28 [95% CI 2.27–461.35]) and a decreased odds for a high LDL/HDL ratio at 6 months (OR 0.16 [95% CI 0.03–0.76]). **Conclusions:** Choline alphoscerate did not show superiority over the placebo in terms of the alleviation of depressive symptoms, despite significant pre-post changes observed within the choline alphoscerate group. Nevertheless, given its positive potential for insulin resistance, the effects of choline alphoscerate on depressive mood in relation to insulin resistance in patients with type 2 diabetes require further validation.

## 1. Introduction

The prevalence of diabetes is increasing globally, and if current trends continue, over 700 million adults will have diabetes by 2045 (a global prevalence of 12.2%) [1]. Among patients with diabetes, depression is a highly comorbid condition [2], with a prevalence rate over three times higher in those with type 1 diabetes and nearly twice as high in those with type 2 diabetes compared with those without diabetes [3]. Depression in patients with diabetes is associated with a risk of diabetic complications and a worse prognosis. Patients with both major depression and diabetes are 1.5–2 times more likely to have cardiovascular risk factors than diabetic patients without depression, irrespective of cardiovascular disease status [4]. Depression is associated with glucometabolic derangement (impaired glucose metabolism and increased insulin resistance) [5,6,7]. Depression is also significantly associated with other diabetic complications, including macrovascular complications, sexual dysfunction, neuropathy, nephropathy, and retinopathy [8]. Mortality was found to be significantly increased in diabetic patients with severe depressive symptoms, determined using the patients’ Centers for Epidemiologic Studies Depression Scale scores compared with those with less severe scores [9]. In addition, even minor depression with type 2 diabetes is associated with 1.7 times higher mortality, and major depression is associated with 2.3 times higher mortality compared with that of a non-depressive status [10]. How depression worsens the severity and prognosis of diabetes could be attributed to poor self-care behaviors, decreased adherence to diabetic diets and medication administration, metabolic abnormalities caused by upregulated counter-regulatory hormones, changes in glucose transport functions, and immunoinflammatory activation [11,12]; therefore, the effective management of depression in patients with diabetes is essential to improve clinical outcomes.

In addition to depression, dementia is another serious neuropsychiatric comorbidity of diabetes [13]. Patients with diabetes had a 1.46 times higher relative risk of Alzheimer’s disease (AD) than patients without diabetes [13]. Dementia and depression are closely related, as 90% of patients with AD are complicated by behavioral and psychological symptoms of dementia, 44% of whom have depression [14]. Mechanistically, disturbances in the cholinergic system are implicated in both AD and depression [15,16,17].

In a meta-analysis of the treatment effect of MDD on neurometabolites, higher choline levels in the frontal lobe were observed in patients with depression post treatment, implying that maintaining higher choline levels might be a therapeutic strategy for MDD [18]. In addition, decreased choline-containing metabolites in the brain amygdala region are associated with the recurrence of depressive episodes during antidepressant maintenance therapy [19]. Gliatamin (choline alphoscerate) is an acetylcholine precursor, and choline alphoscerate is clinically used to treat dementia, demonstrating positive effects in patients with AD [20,21]. In a previous phase 3 clinical trial, choline alphoscerate showed clinical usefulness in the cognitive symptoms of the Alzheimer type-dementia disorders and tolerability [21]. Considering the role of choline metabolism in depressive symptoms, choline alphoscerate may also have beneficial effects on mood disorders such as depression [20]. In another phase 3 clinical trial, patients treated with choline alphoscerate treatment with donepezil showed a significantly lower level of mood disorders than subjects treated with donepezil only [20]. Furthermore, as depression is related to the aggravation of insulin resistance and metabolic derangements, the improvement of depressive symptoms by choline alphoscerate may have metabolically positive effects in patients with diabetes [22].

Thus, in this study, we investigated whether choline alphoscerate can improve depressive symptoms in patients with type 2 diabetes by carrying out a 6-month double-blind, randomized, and placebo-controlled trial.

## 2. Materials and Methods

### 2.1. Study Design

This study was a parallel, double-blind, randomized, and single-center phase 4 clinical trial designed to explore the secondary effects of the drug and identify potential new indications [23,24].

Patients with type 2 diabetes and depressive symptoms from the outpatient clinic of the Endocrinology and Metabolism Department at Severance Hospital were randomly assigned (2:1) to receive choline alphoscerate (800 mg/day) or a placebo for 6 months. A 2:1 allocation was designed considering several strengths, such as improving the ethical balance of the trial, enhancing data collection for the experiment treatment, and aiding in recruitment as well as retention. Randomization was performed using computer-generated random number tables stratified by sex, and the process was executed by a clinical research nurse. Treatment allocation was conducted in a blinded manner using triple masking, involving the participant, care provider, and investigator. Participants received 400 mg of choline alphoscerate or the placebo twice a day with a meal or 30 min after a meal. In a “real world” study of about 3000 major depressive disorder patients, the mean time to response was 5.7 weeks [25]. In our study, we observed the participants for 6 months, and the duration was sufficient to observe significant improvements in depression.

**Figure 1:**
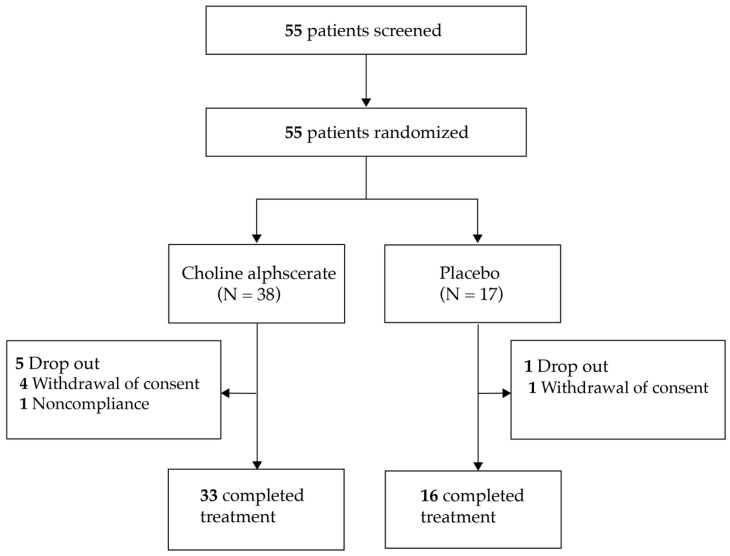
Enrollment, randomization, follow-up, and analysis of the study participants.

The study flows are shown in Figure 1. Screening tests were performed using the Beck Depression Inventory (BDI) to select patients with depressive symptoms of at least mild severity. The BDI is a self-reporting questionnaire that can be used as a screening tool, and responders can be considered as having a mild depressive status with a BDI score ≥ 10 [26,27]. After random assignment, depressive symptom levels were compared between choline alphoscerate and placebo groups.

To objectively evaluate the effect of choline alphoscerate and the placebo on depressive symptoms, the Hamilton Depression Rating Scale (HDRS) was used for the quantification of depressive symptoms [28]. The HDRS is based on a 17-item scale, and the HDRS can be classified as no depression (0–7), mild depression (8–16), moderate depression (17–23), and severe depression (≥24) [29]. An unauthorized version of the Korean Mini-Mental State Examination (MMSE) was used by the study team without permission to evaluate cognitive impairment before and after treatment. The MMSE is a copyrighted instrument and may not be used or reproduced in whole or in part, in any form or language, or by any means without the written permission of PAR (www.parinc.com, accessed on 25 February 2025). After paying the fee for the usage of MMSE, we received a permission letter authorizing the use of the data. MMSE scores are useful for serially documenting cognitive changes and quantitatively estimating the severity of cognitive impairments [30]. MMSE scores of 24–30 are classified as no cognitive impairment, 18–23 as mild cognitive impairment, and 0–17 as severe cognitive impairment [30].

The primary outcome measure was changes in the HDRS after the 6-month treatment. The secondary outcome measure was changes in the MMSE score and glucometabolic parameters after the 6-month treatment.

Telephone monitoring was performed to evaluate adverse events and medication compliance and to check for concomitant medications in the first and third months. We advised the patients to visit the outpatient clinic if necessary or call at any time if they had adverse events. Antidiabetic medication treatment was maintained, and fasting glucose as well as glycated hemoglobin (HbA1c) levels were measured to evaluate the changes in glucose levels. Safety monitoring was conducted during the trial period.

### 2.2. Participants

A total of 55 participants were enrolled, and 49 completed the trial (placebo group = 16 and choline alphoscerate group = 33). Eligible participants were recruited between September 2016 and September 2019, and the final study visit was February 2020.

Eligible criteria for enrollment were as follows: patients with type 2 diabetes aged ≥ 50 years, patients with depressive symptoms (BDI score ≥ 10), patients with glycated hemoglobin (HbA1c) levels ≤ 10.0%, and male or female patients capable of contraception if they were of childbearing age. Participants were excluded if they had type 1 diabetes or other diabetes types such as gestational diabetes mellitus; were taking medication for dementia or depression; had uncontrollable mental diseases; had a BDI score ≥ 30 or severe depressive symptoms; engaged in heavy alcohol consumption (≥210 g/week for men or ≥140 g/week for women); had allergies or hypersensitivity to the medicine or its ingredients; had chronic kidney disease (estimated glomerular filtration rate [eGFR] < 30 mL/min/1.73 m^2^) or were undergoing dialysis treatment; had uncontrollable chronic hepatitis, viral hepatitis (including A, B, or C), liver cirrhosis, or liver cancer; showed abnormal liver function tests (definition: three times or greater increase in the upper limit of normal of aspartate aminotransferase (AST), alanine aminotransferase (ALT), alkaline phosphatase (ALP), or serum total bilirubin levels); had an addiction to alcohol or drugs within 3 months; were breastfeeding or pregnant; or had pancreatitis, pancreatic cancer, or human immunodeficiency virus infection.

This trial was registered in a publicly available registry approved by the International Committee of Medical Journal Editors on ClinicalTrials.gov (registration number: NCT03059069). The Severance Hospital Institutional Review Board approved this study (IRB no. 4–2016–0349), and all participants provided written informed consent.

### 2.3. Clinical Assessments

Sex, age, and height were recorded at the initial visit. Weight, waist circumference (WC), systolic and diastolic blood pressure, and heart rate were measured at each visit. At the first visit, the BDI was used to screen the presence of depressive symptoms. At the second visit and the 6-month third visit after the completion of the trial, the HDRS and MMSE were performed to evaluate the efficacy of drugs. Regarding blood tests, the following parameters were measured: white blood cell count, red blood cell count, hemoglobin, hematocrit, calcium, inorganic phosphate, fasting glucose, postprandial 2 h glucose, blood urea nitrogen, creatinine, uric acid, cholesterol, total protein, albumin, ALP, AST, ALT, total bilirubin, triglycerides, HbA1c, low-density lipoprotein (LDL) cholesterol, high-density lipoprotein (HDL) cholesterol, eGFR calculated by the Chronic Kidney Disease Epidemiology Collaboration (CKD-EPI) equation, and fasting insulin.

### 2.4. Insulin Resistance Measurement

To assess insulin resistance, several parameters were evaluated, including waist circumference, the triglyceride glucose (TyG) index, the TyG index × WC, the LDL/HDL ratio, the ALT/AST ratio [31,32,33,34,35], and homeostasis model assessment for insulin resistance (HOMA-IR) [36]. As the calculation of HOMA-IR includes blood insulin levels [36], which can be influenced by insulin use, the evaluation was also conducted by limiting the participants to non-users of insulin.

### 2.5. Dropout

Participants with a compliance rate of 80% or less were excluded. Compliance was estimated by counting the number of remaining medication capsules. Participants with uncontrolled severe depressive symptoms and suicide attempts due to depressive symptoms were dropped out. Participants who could not complete the trial were excluded from the study. The participants were free to withdraw written consent at any time during the trial, and the reason for withdrawal was documented. If adverse events, including severe ones, occurred, the participants dropped out immediately.

### 2.6. Statistical Analysis

The sample size of this clinical trial was designed to have 90% power (two-sided, α = 0.05, allocation ratio N2/N1 = 2) to test the superiority of choline alphoscerate over a placebo using the G*Power 3.1.9.7. program. A planned minimal sample size of the total study participants was 50 to detect a mean difference in the HDRS of 5.0, assuming a standard deviation (SD) of 5.0. The final sample size was set to 60 participants after assuming a 20% dropout rate.

Continuous variables with normal and non-normal distributions are presented as means with SDs and medians with interquartile ranges, respectively. Categorical variables are presented as numbers with percentages (%). The differences between the treatment groups were evaluated using an independent *t*-test or the Mann–Whitney *U* test for continuous variables with normal and non-normal distributions, respectively. The difference in categorical variables between the treatment groups was calculated using the χ^2^ or Fisher’s exact test, depending on the expected frequency. The significance of changes after 6 months from the baseline for each parameter was determined using paired Student’s *t*-tests or Wilcoxon signed-rank tests in the choline alphoscerate and placebo groups according to the normality of the data distribution. Within each treatment group, analyses were conducted on the changes in and status of insulin resistance-related parameters at 6 months. Improvement in the HDRS was defined as (value at 6 months—value at baseline) <0. Multiple logistic regression analysis was performed to evaluate the adjusted odds ratios (aORs) for the changes in insulin resistance biomarkers after 6 months of the trial. In multiple logistic regression analysis, the LDL/HDL ratio was categorized as low and high, where a high LDL/HDL ratio was defined as the highest tertile group of the LDL/HDL ratio at 6 months, and the remainder was classified as low. Statistical analyses were performed using Statistical Package for the Social Sciences statistical software for Windows (version 25.0; IBM, Armonk, NY, USA). Statistical significance was set to *p* < 0.05.

## 3. Results

### 3.1. Baseline Characteristics

Among the 55 eligible patients, 38 were allocated to the choline alphoscerate group and 17 to the placebo group in a 2:1 ratio. During the follow-up, five patients from the choline alphoscerate group (declined to participate [n = 4], non-compliance [n = 1]) and one from the placebo group (declined to participate) discontinued the study. Finally, 33 patients in the choline alphoscerate group and 16 in the placebo group were analyzed in this study (Figure 1).

At the baseline, despite the difference in participant numbers due to the 2:1 distribution, the choline alphoscerate and placebo groups were well balanced, showing similar clinical and glucometabolic characteristics without significant differences between the two groups (Table 1). The mean (SD) age of the patients was 68.5 (7.8) years; 15 (30.6%) of them were male. The mean (SD) duration of diabetes mellitus was 19.1 (10.7) years, and the median BMI was 23.6 kg/m^2^. Of the patients, 30 (61.2%) had hypertension, 29 (59.2%) had cardiovascular disease, four (8.2%) had cerebrovascular disease, 44 (89.8%) had dyslipidemia, and 12 (24.5%) had a history of psychiatric disorder. The mean (SD) HbA1c was 7.1% (0.9%), and the median HOMA-IR was 3.0 (mg/dL·μIU/mL), suggesting increased insulin resistance (HOMA-IR ≥ 2.5) among the study participants. Patterns of antidiabetic medication use were not different between the two groups. Insulin and biguanides were used in 18 (36.7%) and 42 (85.7%) patients, respectively. The mean baseline HDRS was 15.8 without a significant difference between the two groups, suggesting mild depression at baseline.

### 3.2. Changes in Depressive Symptoms and Glucometabolic Parameters at 6 Months

The changes in neuropsychiatric and glucometabolic parameters after 6 months in the choline alphoscerate and placebo groups are presented in Table 2. There was no change in the Korean-MMSE (K-MMSE) score in either group. The HDRS was significantly decreased in both the choline alphoscerate (15.9 ± 6.5 to 12.6 ± 5.8, *p* = 0.001) and placebo (15.4 ± 4.7 to 10.2 ± 3.9, *p* = 0.004) groups after 6 months, without intergroup differences (*p* = 0.297; Table 2).

Most variables showed no statistically significant intergroup differences in changes from the baseline to 6 months between the placebo and choline alphoscerate groups. Meanwhile, some indices related to insulin resistance demonstrated trends toward more pronounced improvements in the choline alphoscerate group than the placebo group. At 6 months, WC decreased by 0.4 cm from the baseline in the choline alphoscerate group, while it increased by 0.6 cm in the placebo group, demonstrating a significant difference between the two groups (*p* = 0.009; Table 2). For the LDL/HDL ratio, the choline alphoscerate group tended to maintain levels, while the placebo group numerically exhibited an increase. The difference between the two groups was marginally significant (*p* = 0.092; Table 2). HOMA-IR significantly decreased only in the choline alphoscerate group (2.9 to 1.6, *p* = 0.002), even though the change in HOMA-IR at 6 months did not show a significant difference between the two groups (*p* = 0.249; Table 2).

When comparing the proportion of subjects who showed improvement in insulin resistance-related parameters between the placebo and the choline alphoscerate groups (Table S1), the proportion was numerically higher in the choline alphoscerate group, although the majority did not reach statistical significance. Nevertheless, the proportion of participants who showed a reduction in WC was significantly higher in the choline alphoscerate group than the placebo group (39.4% vs. 6.3%, *p* = 0.019). Additionally, the proportion of individuals with a high LDL/HDL ratio at 6 months was statistically lower in the choline alphoscerate group than the placebo group (22.6% vs. 56.3%, *p* = 0.021).

Within each treatment group, analysis based on HDRS improvement showed that in the choline alphoscerate group, the proportion of participants who showed a reduction in WC was numerically higher among those with an improved HDRS compared to those without improvement (47.6% vs. 25.0%, *p* = 0.278, Table S1). In the placebo group, the number of participants who showed a reduction in WC was limited, making it difficult to analyze this pattern based on changes in the HDRS. Additionally, the proportion of individuals with a high WC at 6 months was numerically lower in the choline alphoscerate group than the placebo group, although there was no statistical difference (33.3% vs. 37.5%, *p* = 0.774). In the choline alphoscerate group, those with HDRS improvement had a significantly lower proportion of high WC at 6 months compared to those without improvement (14.3% vs. 66.7%, *p* = 0.005). This trend was not observed in the placebo group.

### 3.3. Choline Alphoscerate Treatment as a Predictive Factor for Favorable Insulin Resistance-Related Parameters

To determine whether choline alphoscerate use was a predictive factor for the changes in and status of insulin resistance-related parameters at 6 months, a multiple logistic regression analysis was performed (Table 3). After adjusting for multiple confounding factors, including age, sex, BMI (≥25 vs. <25 kg/m^2^), changes in depression scores expressed by HDRS change, and insulin use, choline alphoscerate use was associated with increased odds for a reduction in WC (OR 18.28 [95% CI 2.27–461.35]) and decreased odds for a high LDL/HDL ratio at 6 months (OR 0.16 [95% CI 0.03–0.76]). Because age, sex, and BMI are related with insulin resistance and depression, we opted for these factors as variables [37,38,39,40,41,42,43]. Whether the effects of each clinical variable on changes in insulin resistance parameters differ depending on the presence or absence of improvement in the HDRS was further examined (Tables S2 and S3). The OR for improvement in WC by choline alphoscerate use was higher in the improved HDRS group than in the total subjects (ORs 22.69 vs. 14.40; Table S2). In addition, the risk reduction for a high LDL/HDL ratio associated with choline alphoscerate use was also more pronounced in the improved HDRS group than in the total subjects (ORs 0.03 vs. 0.23; Table S3). The effect of choline alphoscerate use on insulin resistance parameters could not be evaluated in the non-improved HDRS group due to complete separation [44]. This was because the small sample size resulted in placebo users within the non-improved HDRS group being biased toward one of the outcome occurrence/non-occurrence categories in the logistic models [44]. Although the small sample size makes it difficult to draw definitive conclusions, the positive impact of choline alphoscerate use on insulin resistance parameters might be more significant when choline alphoscerate improves depression symptoms.

### 3.4. Safety

The proportion of patients with one or more adverse events (AEs) was not significantly different between the two groups (0 [0.0%] vs. three [9.1%] in the placebo and choline alphoscerate group, respectively, *p* = 0.541). One participant discontinued the medication because of heartburn, which led to non-compliance and discontinuation of the study. No severe AEs were observed. Two serious AEs occurred in the choline alphoscerate group (appendicitis and pelvic organ prolapse) which were unrelated to the drug (Table S4).

## 4. Discussion

In this study, the administration of choline alphoscerate (800 mg/day) for 6 months did not lead to superior improvement in depressive symptoms, as assessed by the HDRS, compared to the placebo in older patients with type 2 diabetes; however, considering the placebo’s effect on psychiatric mood disorders, which can even be comparable to the pharmaceutical effect [45,46,47], the effect of choline alphoscerate on depressive symptoms may not be completely ruled out, as choline alphoscerate significantly decreased the HDRS from the baseline within the treatment group. Meanwhile, the choline alphoscerate group showed a significantly more pronounced benefit in insulin resistance parameters, including WC and the LDL/HDL ratio, than the placebo group at 6 months. Choline alphoscerate treatment was an independent predictive factor for a higher likelihood of WC reduction and a low LDL/HDL ratio at 6 months. Thus, our data suggest the potential value of choline alphoscerate, a drug used for AD, for depressive symptoms in relation to metabolic benefits in older adult patients with long-standing type 2 diabetes.

Type 2 diabetes and aging are important clinical factors that reinforce the choline-mediated pathophysiological connection between AD and depression. Comorbid type 2 diabetes in patients with AD was an independent risk factor for the development of depression [48]. Cholinergic dysfunction in AD can be further aggravated by hyperglycemia and insulin resistance attributed to type 2 diabetes [49], which might increase the risk of depression. Aging is also a major contributing factor to choline deficiency in the brain [50], increasing the risk of both dementia and depression. In the present study, the mean age of the patients was approximately 70 years, which was far older than the cut-off age of 50 years required for study inclusion. The higher-than-expected mean age of the patients prior to enrollment could be attributed to the study inclusion criterion of depressive symptoms with BDI scores of 10 or more, given that older adults are at a greater risk of depression than middle-aged individuals [51].

Considering the common pathophysiology of the dysfunctional choline system in AD and depression, choline alphoscerate should be examined for its clinical usefulness in depressive symptoms, particularly in older individuals and those with type 2 diabetes. The beneficial effects of choline alphoscerate on mood disorders have recently been reported in patients with AD [20]. Compared with the administration of donepezil alone, combination therapy with choline alphoscerate significantly improved mood disorders, including depression, anxiety, and apathy [20]. Meanwhile, treatment with an acetylcholine precursor has been shown to induce depression in some previous reports [52,53]; however, these studies were conducted in a limited number of patients, and affected patients had underlying neuropsychiatric abnormalities, such as tardive dyskinesia or a history of affective disorders [52,53]. Therefore, it could be inferred that choline alphoscerate may affect moods differently according to subjects’ underlying neuropsychiatric conditions. To the best of our knowledge, the single effect of choline alphoscerate on depressive symptoms in patients without neuropsychiatric disorders has never been investigated. This is the first study to investigate the effect of choline alphoscerate on depressive symptoms in patients with type 2 diabetes and without neuropsychiatric disorders. Hence, the present data are clinically important, as they can inspire future studies on the effect of choline alphoscerate on mood in the general population with type 2 diabetes.

In the current study, the choline alphoscerate treatment showed benefits for insulin resistance parameters including WC and the LDL/HDL ratio. This may be partly explained by the attenuation of depressive symptoms in the choline alphoscerate group. Depression is associated with the activation of the hypothalamic–pituitary–adrenal (HPA) axis [54]. A hyperactive HPA axis, which causes excessive circulating cortisol levels, disrupts glucoregulatory mechanisms and induces insulin resistance in hyperinsulinemia [54]. High cortisol levels can induce the accumulation of fat, particularly in the intra-abdominal visceral area, which is characterized by central obesity and a high waist circumference [55]. Excessive cortisol is also associated with lipid abnormalities, including high LDL and low HDL [56]. Therefore, the alleviation of depressive symptoms by choline alphoscerate is likely to attenuate excessive cortisol associated with depressive symptoms and subsequent metabolic disruptions; however, the improvement in depression was not statistically different between the choline alphoscerate and placebo groups, likely due to the placebo effect, implying that the amelioration of depressive mood itself cannot fully explain the benefit of choline alphoscerate on insulin resistance. Nevertheless, when analyzed based on the presence or absence of HDRS improvement, only the choline alphoscerate group showed an association between HDRS improvement and WC benefits. Furthermore, only participants with an improved HDRS showed significant associations between choline alphoscerate use and favorable insulin resistance parameters in logistic regression models, even more prominent in the analyses of total participants, including those with a non-improved HDRS. Therefore, although the limited number of subjects did not allow for robust statistical significance, our results suggest the possibility that choline alphoscerate treatment might improve the features of insulin resistance by modulating depressive symptoms.

In addition to the amelioration of depressive symptoms, increased brain levels of acetylcholine [17] and enhanced brain cholinergic signaling by choline alphoscerate [17] might also underlie the mechanism by which choline alphoscerate showed potential advantages in insulin resistance parameters in the present study. The brain controls peripheral metabolic functions in the context of obesity-driven disorders, and neuroinflammation under the milieu of insulin resistance in obesity affects the hypothalamus, hippocampus, amygdala, and cortex, all of which receive cholinergic innervation [57]. Brain cholinergic signaling is involved in the regulation of appetite and feeding behaviors, hepatic glycogen synthesis, pancreatic secretion, and the control of peripheral inflammation through vagus nerve-mediated mechanisms [57]. Thus, restoring diminished brain choline levels in type 2 diabetes may provide metabolic benefits and ameliorate insulin resistance [57,58,59]. The activation of brain cholinergic signaling by an acetylcholine esterase inhibitor could modulate autonomic neural regulation, exhibit anti-inflammatory effects, and alleviate brain as well as systemic insulin resistance [57,58,59]. Central cholinergic activation by galantamine lowered insulin resistance, cholesterol, and abdominal fat depots in vivo [60], which aligns with the association of choline alphoscerate use with WC reduction and low LDL/HDL profiles observed in the current study.

A few limitations must be considered when interpreting the current data. First, this study was conducted with a relatively small number of participants. One significant limitation of small studies is the risk of producing less reliable results [61]. They may be unable to demonstrate statistical significance for genuinely meaningful relationships or, alternatively, may yield false positive results [61]. Achieving reliable statistical analysis through the adjustment of several variables also necessitates an adequate sample size [61]. The improvement in depressive symptoms was not significantly superior in the choline alphoscerate group than the placebo group. This could be due to the insufficient number of study participants. We assumed a mean difference in the HDRS of 5.0 and a standard deviation (SD) of 5.0 for calculating the sample size, but in the result of our study, the mean difference in the HDRS was less than 5.0. Like this, a larger sample size is needed to detect the difference when the real effect size is smaller than the expected effect size. In addition, the wide confidence interval in Table 2 also suggests that the sample size was not enough. Therefore, there is a good chance that a type II statistical error exists. Nevertheless, small randomized controlled trials (RCTs) are time- and cost-efficient and reduce participants’ exposure to potential risks from exploring unproven hypotheses [61,62]. In addition, the research findings from a small RCT can serve as a basis for conducting more extensive studies [61,62]. Such consecutive research steps, progressing from smaller- to larger-scale studies, have also been observed in the previous development of antidepressive medication [63]. Thus, despite the limitations of a small sample size, the current study still provides a foundation for future studies on larger scales and longer durations, investigating the effect of choline alphoscerate on depressive symptoms in individuals with type 2 diabetes. Second, a mild degree of depressive symptoms (HDRS around 16 at the baseline) in the study participants might have mitigated the superiority of choline alphoscerate on depressive mood. In recent meta-analyses, the placebo effect accounted for 67% of the real pharmaceutical effect in patients receiving antidepressants, and the placebo was as effective as antidepressants in patients with mild-to-moderate major depression (HDRS < 25), whereas the placebo was less effective than antidepressants in severely depressed patients [64]. Further studies should include patients with varying spectra of depression to verify the effects of choline alphoscerate on depressive moods. Third, lifestyle habits, such as diet, alcohol consumption, and exercise, could not be completely controlled, which may have confounded the results by leading to mood and metabolic changes in both groups. To determine the mechanisms that explain the positive impact of the choline alphoscerate group on insulin resistance parameters, measuring more factors, including appetite, feeding behaviors, C-reactive protein, and lifestyle habits, is required in further studies. Fourth, cognitive function was evaluated considering the original use of choline alphoscerate as a drug for AD; however, all participants had normal cognitive function at the baseline, and no change in cognitive function was observed in either the choline alphoscerate or placebo group. Thus, the effect of choline alphoscerate on cognitive function associated with depression could not be confirmed in the current study.

In conclusion, choline alphoscerate did not demonstrate a greater advantage in depressive symptoms over the placebo in older individuals with type 2 diabetes, although there was a significant improvement in depressive symptoms from the baseline with choline alphoscerate use. On the other hand, the choline alphoscerate group was found to have a significantly positive impact on several insulin resistance parameters after 6 months compared to the placebo group as well to show safety. Future studies including an extended number of patients with various degrees of depressive symptoms are warranted to confirm the neurometabolic effects of choline alphoscerate in older patients with type 2 diabetes and comorbid depression.

## Figures and Tables

**Table 1:** Baseline clinical characteristics of the patients.

	Total (n = 49)	Placebo Group (n = 16)	Choline Alphoscerate Group (n = 33)	*p* Value
HDRS	15.8 (5.9)	15.4 (4.7)	15.9 (6.5)	0.758
K-MMSE	30 (29, 30)	30 (29, 30)	30 (29, 30)	0.999
Age (years)	68.5 (7.8)	68.8 (8.5)	68.4 (7.5)	0.862
Sex (male, n, %)	15 (30.6)	5 (31.2)	10 (30.3)	0.999
DM duration (years)	19.1 (10.7)	15.4 (9.7)	20.9 (10.8)	0.087
Height (cm)	157.8 (9.9)	158.3 (9.3)	157.6 (10.3)	0.812
Weight (kg)	61.9 (11.2)	63.9 (11.2)	60.9 (11.2)	0.394
BMI (kg/m^2^)	23.6 (22.5, 26.9)	25.7 (22.8, 27.4)	23.5 (22.4, 25.4)	0.228
Waist circumference (cm)	89.3 (8.6)	89.8 (8.9)	89.0 (8.6)	0.748
Hypertension (n, %)	30 (61.2)	7 (43.8)	23 (69.7)	0.151
Cardiovascular disease (n, %)	29 (59.2)	9 (56.2)	20 (60.6)	0.999
Cerebrovascular disease (n, %)	4 (8.2)	3 (18.8)	1 (3.0)	0.184
Dyslipidemia (n, %)	44 (89.8)	13 (81.2)	31 (93.9)	0.383
Psychiatric disorder (n, %)	12 (24.5)	3 (18.8)	9 (27.3)	0.726
SBP (mmHg)	125.1 (11.1)	124.8 (13.7)	125.2 (9.9)	0.893
DBP (mmHg)	72.6 (7.8)	75.7 (9.2)	71.1 (6.7)	0.055
HbA1c (%)	7.1 (0.9)	6.8 (0.9)	7.3 (0.9)	0.082
Glycoalbumin (%)	18.7 (3.3)	18.2 (3.5)	19.1 (3.3)	0.579
Fasting glucose (mg/dL)	132.0 (110.0, 152.0)	122.5 (106.2, 153.5)	133 (113.0, 142.0)	0.468
Postprandial 2 h glucose (mg/dL)	212.0 (168.0, 251.5)	205.0 (149.0, 219.5)	224.0 (178.5, 263.8)	0.247
Fasting insulin (μIU/mL)	8.3 (5.1, 16.7)	10.4 (7.2, 13.5)	6.6 (5.0, 17.9)	0.316
HOMA-IR (mg/dL·μIU/mL) in all subjects	3.0 (1.6, 5.4)	3.2 (2.4, 4.7)	2.9 (1.5, 5.4)	0.633
HOMA-IR (mg/dL·μIU/mL) in insulin non-users (n = 31)	2.8 (1.6, 4.9)	3.1 (2.6, 5.0)	2.0 (1.6, 3.8)	0.227
TyG index	8.9 (0.6)	8.9 (0.7)	8.9 (0.6)	0.977
TyG index × waist circumference (cm)	795.0 (93.0)	799.0 (88.9)	793.0 (96.2)	0.837
Triglyceride (mg/dL)	126.5 (59.1)	137.8 (63.0)	121.1 (57.3)	0.360
HDL cholesterol (mg/dL)	50.7 (10.0)	46.9 (8.6)	52.6 (10.2)	0.061
LDL cholesterol (mg/dL)	84.5 (28.3)	90.3 (36.3)	81.7 (23.6)	0.322
LDL/HDL ratio	1.7 (0.7)	2.0 (0.9)	1.6 (0.6)	0.089
eGFR (CKD-EPI, mL/min/1.73 m^2^)	88.0 (72.0, 96.0)	89.0 (78.8, 95.0)	86.0 (69.0, 96.0)	0.654
AST (IU/L)	20.0 (18.0, 24.0)	23.0 (18.8, 26.8)	19.0 (17.0, 23.0)	0.080
ALT (IU/L)	18.0 (13.0, 25.0)	19.0 (13.0, 26.2)	18.0 (0.7, 1.1)	0.717
ALT/AST ratio	0.8 (0.7, 1.1)	0.8 (0.6, 1.1)	0.9 (0.7, 1.1)	0.488
Antidiabetic medication use at baseline				
Insulin (n, %)	18 (36.7)	5 (31.2)	13 (39.4)	0.811
Biguanides (n, %)	42 (85.7)	14 (87.5)	28 (84.8)	0.999
Sulfonylureas (n, %)	21 (42.9)	6 (37.5)	15 (45.5)	0.826
DPP-4i (n, %)	24 (49.0)	9 (56.2)	15 (45.5)	0.686
TZD (n, %)	10 (20.4)	1 (6.2)	9 (27.3)	0.182
SGLT2i (n, %)	4 (8.2)	1 (6.2)	3 (9.1)	0.999
αGI (n, %)	3 (6.1)	1 (6.2)	2 (6.1)	0.999

Values are shown as numbers (%), mean (SD), or median (interquartile range). HDRS, Hamilton Depression Rating Scale; K-MMSE, Korean Mini-Mental State Examination; DM, diabetes mellitus; BMI, body mass index; SBP, systolic blood pressure; DBP, diastolic blood pressure; HbA1c, glycated hemoglobin; HOMA-IR, homeostasis model assessment for insulin resistance; TyG index, triglyceride glucose index; HDL, high-density lipoprotein; LDL, low-density lipoprotein; eGFR, estimated glomerular filtration rate; CKD-EPI, Chronic Kidney Disease Epidemiology Collaboration; AST, aspartate aminotransferase; ALT, alanine aminotransferase; DPP-4i, Dipeptidyl Peptidase-4 inhibitor; TZD, thiazolidinedione; SGLT2i, sodium–glucose transport protein 2 inhibitor; αGI, alpha-glucosidase inhibitor.

**Table 2:** Changes in neuropsychiatric and glucometabolic parameters after 6 months of the placebo or choline alphoscerate treatment.

	Placebo Group (n = 16)				Choline Alphoscerate Group (n = 33)				Intergroup Difference for Post-Pre Values	*p* Value for Intergroup Difference in Post-Pre Values
	Pre 0 M	Post 6 M	Post-Pre (6 M-0 M)	*p* Value	Pre	Post 6 M	Post-Pre (6 M-0 M)	*p* Value		
HDRS	15.4 (4.7)	10.2 (3.9)	−5.2 (6.1)	0.004	15.9 (6.5)	12.6 (5.8)	−3.4 (5.5)	0.001	1.8 (−1.7, 5.3)	0.297
K-MMSE	30 (29, 30)	30 (29, 30)	0.0 (0.0, 0.0)	0.999	30 (29, 30)	30 (29, 30)	0.0 (0.0, 0.0)	0.355	0.1 (−0.3, 0.6)	0.576
BMI (kg/m^2^)	25.7 (22.6, 27.1)	25.9 (22.6, 27.1)	0.0 (−0.2, 0.5)	0.562	23.5 (22.4, 25.4)	23.2 (22.1, 25.2)	−0.1 (−0.5, 0.3)	0.287	−0.2 (−0.5, 0.1)	0.247
WC (cm)	89.8 (8.9)	90.5 (9.1)	0.6 (0.9)	0.016	89.0 (8.6)	88.5 (8.9)	−0.4 (1.4)	0.083	−1.1 (−1.8, −0.3)	0.009
SBP (mmHg)	124.8 (13.7)	125.1 (10.9)	0.4 (11.8)	0.901	125.2 (9.9)	123.1 (10.3)	−2.2 (11.8)	0.303	−2.5 (−9.8, 4.7)	0.486
DBP (mmHg)	75.7 (9.2)	75.8 (9.1)	0.1 (10.7)	0.964	71.1 (6.7)	71.4 (8.0)	0.3 (9.1)	0.864	0.1 (−5.8, 6.1)	0.960
HbA1c (%)	6.8 (0.9)	6.9 (0.8)	0.1 (0.4)	0.276	7.3 (0.9)	7.5 (1.3)	0.2 (0.8)	0.164	0.1 (−0.4, 0.5)	0.667
Glycoalbumin (%)	18.2 (3.5)	19.1 (3.9)	0.4 (2.6)	0.751	19.1 (3.3)	22.1 (5.4)	1.5 (4.2)	0.381	1.1 (−3.7, 5.9)	0.618
FBG (mg/dL)	122.5 (106.2, 153.5)	123.5 (107.5–171.2)	2.5 (−4.0, 33.0)	0.232	133.0 (113.0–142.0)	133.0 (114.0–147.0)	−1.0 (−12.0, 7.0)	0.575	−17.9 (−40.2, 4.5)	0.114
PPG-2h (mg/dL)	205.0 (149.0–219.5)	194.0 (126.0, 214.0)	1.5 (−27.2, 36.5)	0.909	224.0 (178.5, 263.8)	215.5 (181.5, 295.5)	−5.5 (−28.5, 40.0)	0.733	9.9 (−41.6, 61.4)	0.698
Fasting insulin (μIU/mL)	10.4 (7.2, 13.5)	7.5 (3.9, 13.0)	−2.7 (−8.5, 1.7)	0.144	6.6 (5.0, 17.9)	5.1 (3.3, 7.2)	−1.7 (−10.5, 0.2)	0.001	−3.3 (−11.5, −4.8)	0.417
HOMA-IR (mg/dL·μIU/mL) (total subjects)	3.2 (2.4–4.7)	2.4 (0.9, 4.1)	−0.5 (−2.8, 0.5)	0.212	2.9 (1.5–5.4)	1.6 (0.9–2.4)	−0.6 (−3.5, 0.2)	0.002	−1.8 (−4.8, 1.3)	0.249
HOMA-IR (mg/dL·μIU/mL) (insulin non-users only, n = 31)	3.1 (2.6, 5.0)	2.4 (2.1, 3.9)	−0.5 (−2.4, 0.7)	0.278	2.0 (1.6, 3.8)	1.5 (1.1, 2.3)	−0.5 (−1.8, 0.3)	0.043	−1.1 (−5.2, 3.0)	0.595
TyG index	8.9 (0.7)	9.1 (0.5)	0.2 (0.6)	0.302	8.9 (0.6)	8.9 (0.6)	0.0 (0.5)	0.665	−0.1 (−0.5, 0.2)	0.425
TyG index × WC (cm)	799.0 (88.9)	820.6 (83.6)	21.7 (55.3)	0.137	793.0 (96.2)	792.4 (98.2)	−0.7 (51.8)	0.940	−22.3 (−54.8, 10.1)	0.173
Triglyceride (mg/dL)	137.8 (63.0)	139.3 (40.9)	1.6 (71.7)	0.931	121.1 (57.3)	138.5 (101.9)	17.4 (90.3)	0.277	15.8 (−36.2, 67.8)	0.544
HDL-C (mg/dL)	46.9 (8.6)	45.2 (10.0)	−1.6 (5.4)	0.244	52.6 (10.2)	49.6 (12.1)	−3.0 (9.1)	0.071	−10.8 (−24.3, 2.6)	0.590
LDL-C (mg/dL)	90.3 (36.3)	98.7 (40.5)	8.5 (19.6)	0.104	81.7 (23.6)	78.6 (26.0)	−2.4 (22.7)	0.563	−1.3 (−6.3, 3.6)	0.111
LDL/HDL ratio	2.0 (0.9)	2.2 (0.9)	0.3 (0.4)	0.014	1.6 (0.6)	1.6 (0.6)	0.0 (0.5)	0.623	−0.2 (−0.5, 0.0)	0.092
ALT/AST ratio	0.8 (0.6, 1.1)	0.8 (0.7, 1.1)	0.0 (−0.2, 0.1)	0.979	0.9 (0.7, 1.1)	0.9 (0.7, 1.1)	0.0 (−0.1, 0.1)	0.817	0.2 (−0.1, 0.5)	0.240

Values are shown as numbers (%), mean (SD), or median (interquartile range). Intergroup difference was presented as the mean with a 95% confidence interval. M, months; HDRS, Hamilton Depression Rating Scale; K-MMSE, Korean Mini-Mental State Examination; BMI, body mass index; WC, waist circumference; SBP, systolic blood pressure; DBP, diastolic blood pressure; HbA1c, glycated hemoglobin; FBG, fasting blood glucose; PPG-2h, 2 h post prandial glucose; HOMA-IR, homeostasis model assessment for insulin resistance; TyG index, triglyceride glucose index; HDL-C, high-density lipoprotein cholesterol; LDL-C, low-density lipoprotein cholesterol; ALT, alanine aminotransferase; AST, aspartate aminotransferase.

**Table 3:** Choline alphoscerate treatment as a determinant factor for changes in insulin resistance-related parameters at 6 months.

n = 49	Improvement in WC ^#^		High LDL/HDL Ratio * at 6 Months	
	OR (95% CI)	*p* Value	OR (95% CI)	*p* Value
Age (years)	0.89 (0.79–0.99)	**0.033**	1.03 (0.93–1.15)	0.568
Sex (female vs. male)	2.68 (0.52–17.87)	0.264	11.14 (1.54–178.48)	0.041
BMI (≥25 vs. <25 kg/m^2^)	1.12 (0.20–6.50)	0.897	0.81 (0.15–4.08)	0.798
Changes in depression scores expressed by HDRS change ^§^	1.00 (0.87–1.16)	0.981	0.88 (0.75–1.02)	0.106
Insulin use	0.27 (0.03–1.65)	0.190	4.95 (0.85–38.92)	0.094
Choline alphoscerate vs. placebo	18.28 (2.27–461.35)	**0.022**	0.16 (0.03–0.76)	**0.029**

^#^ Improvement in WC was defined as the value at (6 months—value at the baseline) <0. * A high LDL/HDL ratio was defined as the highest tertile group of the LDL/HDL ratio at 6 months. ^§^ Changes in depression scores expressed by HDRS change were defined as value at 6 months—value at the baseline. A multiple logistic regression analysis was performed. Bold represents statistically significant values (*p* < 0.05). WC, waist circumference; LDL, low-density lipoprotein cholesterol; HDL, high-density lipoprotein cholesterol; OR, odds ratio; 95% CI, 95% confidence interval; BMI, body mass index; HDRS, Hamilton Depression Rating Scale.

## Data Availability

The data that support the findings of this study are not publicly available due to containing information that could compromise the privacy of research participants but are available from the corresponding author upon reasonable request.

## Supplementary Materials

The following supporting information can be downloaded at: https://www.mdpi.com/article/10.3390/jcm14051664/s1, Table S1. Insulin resistance parameters at 6 months according to HDRS improvement in the placebo and choline alphoscerate groups [65]. Table S2. Choline alphoscerate treatment as a determinant factor for changes in insulin resistance-related parameter (WC) at 6 months. Table S3. Choline alphoscerate treatment as a determinant factor for changes in insulin resistance-related parameter (LDL/HDL ratio) at 6 months. Table S4. Adverse events.
