# Supplementary material for: Potential Impact of Choline Alphoscerate on Depressive Symptoms in Association with Insulin Resistance in Elderly Patients with Type 2 Diabetes
Source: J Clin Med. 2025 Feb 28;14(5):1664. doi: 10.3390/jcm14051664 (PMC11900303; doi:10.3390/jcm14051664)
Supplement: Supplementary file 1 [file jcm-14-01664-s001.zip › jcm-3445806 Table S1.pdf]

Table S1. Insulin resistance parameters at 6 months according to HDRS improvement in the placebo and choline alphoscerate groups.

	Placebo group				Choline alphoscerate group				
	Total (n = 16)	Non-improvement in HDRS (n = 4)	Improvement in HDRS[#] (n = 12)	*p* value	Total (n = 33)	Non-improvement in HDRS (n =12)	Improvement in HDRS[#] (n = 21)	*p* value	*p* value for intergroup difference at 6 months
WC improvement[#]	**1 (6.3)**	0 (0.0)	1 (8.3)	> 0.999	**13 (39.4)**	3 (25.0)	10 (47.6)	0.278	**0.019**
Abdominal obesity (WC ≥ 90 cm in men and ≥ 85 cm in women) [1]	12 (75.0)	4 (100.0)	8 (66.7)	0.516	20 (60.6)	8 (66.7)	12 (57.1)	0.719	0.360
High WC*	6 (37.5)	2 (50.0)	4 (33.3)	0.604	11 (33.3)	**8 (66.7)**	**3 (14.3)**	**0.005**	0.774
TyG index improvement[#]	5 (31.3)	1 (25.0)	4 (33.3)	> 0.999	17 (51.5)	7 (8.3)	10 (47.6)	0.895	0.181
High TyG index*	7 (43.8)	2 (50.0)	5 (41.7)	> 0.999	10 (30.3)	4 (33.3)	6 (28.6)	> 0.999	0.354
TyG index × WC improvement[#]	4 (25.0)	1 (25.0)	3 (25.0)	> 0.999	16 (48.5)	6 (50.0)	10 (47.6)	0.895	0.117
High TyG × WC index*	7 (43.8)	2 (50.0)	5 (41.7)	> 0.999	10 (30.3)	5 (41.7)	5 (23.8)	0.433	0.354
LDL/HDL ratio improvement[#]	5 (31.3)	2 (50.0)	3 (25.0)	0.547	12 (38.7)	6 (50.0)	6 (31.6)	0.452	0.614
High LDL/HDL ratio*	**9 (56.3)**	**0 (0.0)**	**9 (75.0)**	**0.019**	**7 (22.6)**	4 (33.3)	3 (15.8)	0.384	**0.021**
ALT/AST ratio improvement[#]	7 (43.8)	2 (50.0)	5 (41.7)	> 0.999	19 (59.4)	5 (45.5)	14 (66.7)	0.283	0.306
HOMA-IR improvement[#]	11 (68.8)	2 (50.0)	9 (75.0)	0.547	20 (66.7)	9 (75.0)	11 (61.1)	0.694	0.886
High HOMA-IR (≥ 2.5)	7 (43.8)	2 (50.0)	5 (41.7)	> 0.999	6 (20.0)	2 (16.7)	4 (22.2)	> 0.999	0.167

Values are shown as numbers (%). [#]Improvement in parameters was defined as (value at 6 months − value at baseline) < 0. *High WC, TyG, TyG × WC, LDL/HDL, and ALT/AST ratio were defined as the highest tertile group of each parameter at 6 months. HDRS, Hamilton Depression Rating Scale; WC, waist circumference; TyG index, Triglyceride Glucose index; LDL, low-density lipoprotein cholesterol; HDL, high-density lipoprotein cholesterol; ALT, alanine aminotransferase; AST, aspartate aminotransferase; HOMA-IR, homeostasis model assessment for insulin resistance.
