# Supplementary material for: Potential Impact of Choline Alphoscerate on Depressive Symptoms in Association with Insulin Resistance in Elderly Patients with Type 2 Diabetes
Source: J Clin Med. 2025 Feb 28;14(5):1664. doi: 10.3390/jcm14051664 (PMC11900303; doi:10.3390/jcm14051664)
Supplement: Supplementary file 1 [file jcm-14-01664-s001.zip › jcm-3445806 Table S2.pdf]

Table S2. Choline alphoscerate treatment as a determinant factor for changes in insulin resistance-related parameter (WC) at 6 months.

	**Improvement in WC[#]**					
n = 49	Total		Improved HDRS[#] (n = 33)		Non-improved HDRS (n = 16)	
	OR (95% CI)	***p* value**	OR (95% CI)	***p* value**	OR (95% CI)	***p* value**
Age (years)	0.89 (0.79–0.99)	**0.029**	0.83 (0.68–0.97)	**0.033**	0.91 (0.70–1.11)	0.364
Sex (female vs. male)	2.41 (0.45–12.82)	0.301	14.45 (1.20–507.51)	0.070	0.00 (NA–3.92×10^{304})	0.997
BMI (≥ 25 vs. < 25 kg/m²)	0.77 (0.16–3.63)	0.736	0.66 (0.07–5.20)	0.696	2.65×10^{8} (0.00–NA)	0.998
Changes in HDRS[§]	0.97 (0.85–1.11)	0.693	−	−	−	−
Choline alphoscerate vs. Placebo	14.40 (1.25–165.3)	**0.032**	22.69 (1.99–1063.21)	**0.039**	4.90×10^{16} (0.00–NA)	0.999

[#]Improvement in WC and HDRS was defined as value at (6 months − value at the baseline) < 0. [§]Changes in HDRS were defined as value at 6 months − value at the baseline. A multiple logistic regression analysis was performed. Bolds represent statistically significant values ($p < 0.05$). Due to complete separation, the confidence interval estimates are extremely wide in improved HDRS and non-improved HDRS. HDRS, Hamilton Depression Rating Scale; WC, waist circumference; OR, odds ratio; 95% CI, 95% confidence interval; NA, Not applicable; BMI, body mass index.
