# Supplementary material for: Potential Impact of Choline Alphoscerate on Depressive Symptoms in Association with Insulin Resistance in Elderly Patients with Type 2 Diabetes
Source: J Clin Med. 2025 Feb 28;14(5):1664. doi: 10.3390/jcm14051664 (PMC11900303; doi:10.3390/jcm14051664)
Supplement: Supplementary file 1 [file jcm-14-01664-s001.zip › jcm-3445806 Table S3.pdf]

Table S3. Choline alphoscerate treatment as a determinant factor for changes in insulin resistance-related parameter (LDL/HDL ratio) at 6 months.

	**High LDL/HDL ratio* at 6 months**					
n = 49	Total		Improved HDRS[#] (n = 33)		Non-improved HDRS (n = 16)	
	OR (95% CI)	***p* value**	OR (95% CI)	***p* value**	OR (95% CI)	***p* value**
Age (years)	1.03 (0.93–1.14)	0.538	1.01 (0.83–1.17)	0.938	1.01 (0.86–1.20)	0.897
Sex (female vs. male)	5.73 (0.92–35.83)	0.062	17.89 (1.27–795.14)	0.065	1.50 (0.05–47.2)	0.795
BMI (≥ 25 vs. < 25 kg/m^2)	1.39 (0.32–5.96)	0.657	1.36 (0.13–15.66)	0.790	8.14 (0.51–322)	0.170
Changes in HDRS[§]	0.91 (0.80–1.05)	0.206	–	–	–	–
Choline alphoscerate vs. Placebo	0.23 (0.05–0.99)	**0.048**	0.03 (0.00–0.23)	**0.004**	1.56×10^8 (0.00–NA)	0.995

*A high LDL/HDL ratio was defined as the highest tertile group of the LDL/HDL ratio at 6 months. [#]Improved HDRS was defined as value at (6 months − value at the baseline) < 0. [§]Changes in HDRS were defined as value at 6 months − value at the baseline. A multiple logistic regression analysis was performed. Bolds represent statistically significant values ($p < 0.05$). Due to complete separation, the confidence interval estimates are extremely wide in improved HDRS and non-improved HDRS. HDRS, Hamilton Depression Rating Scale; LDL, low-density lipoprotein cholesterol; HDL, high-density lipoprotein cholesterol; OR, odds ratio; 95% CI, 95% confidence interval; BMI, body mass index; NA, Not applicable.
