# Supplementary material for: Potential Impact of Choline Alphoscerate on Depressive Symptoms in Association with Insulin Resistance in Elderly Patients with Type 2 Diabetes
Source: J Clin Med. 2025 Feb 28;14(5):1664. doi: 10.3390/jcm14051664 (PMC11900303; doi:10.3390/jcm14051664)
Supplement: Supplementary file 1 [file jcm-14-01664-s001.zip › jcm-3445806 Table S4.pdf]

Table S4. Adverse Events.

	Placebo group (n = 16)	Choline alphoscerate group (n = 33)
**One or more AEs** (n, %)		
Hypoglycemia*	0 (0)	0 (0)
Gastrointestinal disorder	0 (0)	2 (6.1)
Cardiovascular disorder	0 (0)	0 (0)
Nervous system disorder	0 (0)	0 (0)
Genitourinary disorder	0 (0)	1 (3.0)
Neoplasm	0 (0)	0 (0)
Eye disorder	0 (0)	0 (0)
Musculoskeletal disorder	0 (0)	0 (0)
Skin disorder	0 (0)	0 (0)
Kidney disorder	0 (0)	0 (0)
Edema	0 (0)	0 (0)
Insomnia	0 (0)	0 (0)
Weight loss	0 (0)	0 (0)
**One or more drug-related AEs**[#] (n, %)	0 (0)	0 (0)
**AEs leading to discontinuation** (n, %)	0 (0)	1 (3.0)
**One or more severe AEs** (n, %)	0 (0)	0 (0)
**One or more serious AEs** (n, %)	0 (0)	2 (6.1)

*Documented symptomatic (plasma glucose level 70 mg/dL with typical symptoms of hypoglycemia) or asymptomatic (plasma glucose level 70 mg/dL without typical symptoms) hypoglycemia were all recorded. [#]As assessed by the investigator. AEs, adverse events.
